# Shifting from postauricular to transcanal microscopic tympanoplasty may have similar frequency-specific improvements with better air-bone-gap closure at low frequencies and a minimal learning-curve effect

**DOI:** 10.1371/journal.pone.0253947

**Published:** 2021-07-08

**Authors:** Ethan I. Huang, Yu-Chieh Wu, Hsiu-Mei Chuang, Tzu-Chi Huang

**Affiliations:** 1 Department of Otolaryngology, Chang Gung Memorial Hospital, Chiayi, Taiwan; 2 School of Medicine, Chang Gung University, Taoyuan, Taiwan; 3 Audiology and Speech Pathology Center, Chang Gung Memorial Hospital, Chiayi, Taiwan; Sapienza University of Rome, ITALY

## Abstract

The shift from postauricular to transcanal microscopic tympanoplasty brings potential advantages of minimal morbidity, less postoperative pain, patient comfort, and surgical ease and speed, but also uncertainties of unfamiliar grafting material, an inadequate operation view, and an uncertain learning curve. These challenges might affect the successful repair rate and the frequency-specific hearing outcome, which is important for hearing perception. Rare studies reported frequency-specific hearing outcome with the learning curve for shifting from postauricular to transcanal microscopic tympanoplasty. Here, from Jul. 2013 to Nov. 2018, we compared patients in a shift from postauricular approach (35 ears) to transcanal approach (35 ears) of microscopic type-1 tympanoplasty. The results show that both of postauricular and transcanal microscopic tympanoplasties reduced the mean air-bone gap, 0.5k Hz gap, and 1k Hz gap after the surgery. The further analyses on gap change as a function of frequency (0.5, 1, 2, and 4k Hz) show that both of postauricular and transcanal tympanoplasties improved postoperative air-bone gap among the levels of frequency. The post hoc comparisons display a common gap reduction difference between 0.5k and 4k Hz. The successful repair rate did not differ between the 2 groups. There was no correlation between the postoperative mean gap change and the surgery date, suggesting a minimal learning-curve effect. The results of similar frequency-specific improvements and a minimal learning-curve effect may help to ease the concerns of those uncertainties before the shift.

## Introduction

A transcanal tympanoplasty theoretically offers the advantages of minimal morbidity, less postoperative pain [1], patient comfort, and surgical ease and speed [2]. In contrast to a postauricular approach, it can be an outpatient surgery. Patients had a more desirable cosmetic outcome than those undergoing postauricular approaches [3]. Postauricular incision causes not only postoperative pain but also auricular deformity and numbness of the ear [3]. An experienced ear surgeon shifting from postauricular to transcanal technique may face several challenges, such as an unfamiliar grafting material, an inadequate operation view, and an uncertain learning curve. These challenges might affect the frequency-specific hearing outcome.

Grafting material in an exclusive transcanal tympanoplasty may be obtained from or around the tragus cartilage, including cartilage, perichondrium, or soft tissue. This unfamiliar grafting material may be the first challenge an experienced postauricular ear surgeon faces. Cartilage tympanoplasty might show a worse air-bone gap closure at high frequencies [4]. This can result from stiffness or mass effect of a relatively more rigid and thick tympanic membrane reconstructed with cartilage, causing loss of acoustic transfer, especially for high frequencies [4]. Bozdemir et al. reported the pure tone thresholds at the frequencies of 0.5, 1 and 2 k Hz recovered better with temporalis fascia compared to cartilage [5]. Few studies in the literature compared frequency-specific hearing outcome between perichondrium and temporalis-fascia tympanoplasties. On the successful repair rate, most of these comparisons reported no difference between grafting with the perichondrium and temporalis fascia (e.g., see [6]).

Inadequate operation view might be another challenge while shifting to a transcanal technique. Sometimes, a view seeing the whole tympanic membrane or the entire perforation may be impossible either due to overhanging of the bony canal, a narrow canal, or a tortuous canal [7].

An uncertain learning curve is another concern. The lack of experience in the alternative approach may raise the risk linked to professional responsibility [8]. We could not find a report of the learning curve shifting from postauricular to transcanal microscopic tympanoplasty.

On the contrary, several studies investigated the learning curve shifting from postauricular microscopic to transcanal endoscopic tympanoplasty [1,9–11]. The results were diverse. Gokgoz et al. suggested a fast-learning curve [9]. But studies suggested an ear surgeon may need 50 endoscopic patients to achieve significant progress on successful repair rate [10] or approximately 60 operations to master the endoscopic technique [11].

These uncertainties might affect the hearing and repair outcome and whether the above theoretical benefits of transcanal microscopic technique can be achieved. Studies had shown that transcanal and postauricular tympanoplasties resulted in similar average hearing improvement by tympanoplasty without ossiculoplasty [12,13]. But frequency-specific hearing studies are more important because the average pure tone threshold does not directly correlate to patterns of hearing perception [14]. Frequency-specific hearing outcome with the learning curve for shifting from postauricular to transcanal microscopic tympanoplasty has rarely been quantitatively reported. In May 2015, we started to shift microscopic type-1 tympanoplasty with no ossiculoplasty and no mastoidectomy from an admission 3-day postauricular approach using temporalis fascia (postauricular group) to an outpatient transcanal surgery using tragus perichondrium (transcanal group). The purpose of this study was to investigate the effect of the shift for an experienced ear surgeon on successful repair rate and individual frequency-specific hearing outcome.

It is difficult, if not impossible, to randomize assign, double blind, and control the underlying pathologies in the two groups for this purpose. A known underlying pathology is not necessarily linked to a specific hearing outcome [15]. Our earlier work [16] and that of Szaleniec, J. et al [17] has shown that bone-conduction threshold and air-bone gap may predict frequency-specific air-conduction threshold after tympanoplasty. The hearing before surgery is associated with hearing after surgery, regardless of anatomy [18]. Underlying pathologies can represent in bone conduction threshold and air-bone gap [19–23], such as frequency of otorrhea [20,24], size of perforations [20,23,24], infection conditions of the middle ear [19,20], and iatrogenic pathologies (e.g., complications of surgical procedures and simple placement of a ventilation tube [25]). These may be associated with the extent and duration of pathologic change in the middle ear [20–22]. Age, preoperative bone conduction, and preoperative air-bone gap differences were compared to exclude bias of underlying pathologies between the groups. Then, mean gap change and individual gap changes were tested at the frequencies of 0.5k, 1k, 2k, and 4k Hz before and after the surgery for each group. Analyses were taken to show if postoperative gap changes were similar across frequencies, and pair-wise comparisons were conducted to show where a significance existed. To find a possible learning curve, individual postoperative gap changes vs. the surgery dates were scatter plotted, and the correlation was calculated. The difference of successful repair rate between the 2 groups was also investigated.

## Materials and methods

This is a retrospective cohort study. In May 2015, we started to perform type-1 tympanoplasty for all patients by transcanal microscopic approach, unless a patient took the initiative and insisted to receive postauricular approach in the counseling before the surgery (e.g., for insurance payback that requires admission and a specific approach). The enrollment period was between Jul. 2013 and Nov. 2018. So, we reviewed the medical records starting from 2013 to see how many qualified the enrollment criteria for the postauricular group, then matched them with the same number for the transcanal group. Patients receiving type-I tympanoplasty were enrolled in the postauricular group with these criteria:

- Undergoing postauricular microscopic approach
- Using temporalis fascia for grafting
- Having preoperative and postoperative air and bone pure-tone audiometries at 0.5k, 1k, 2k, and 4k Hz

There were total 35 surgeries registered in the postauricular group. Within the same period, the first 35 ears receiving type-I tympanoplasty that met the following but not the exclusion criteria were enrolled in the transcanal group:

- Undergoing transcanal microscopic approach
- Using anterior tragus perichondrium for grafting
- Having preoperative and postoperative air and bone pure-tone audiometries at 0.5k, 1k, 2k, and 4k Hz

The same otolaryngologist, Huang, conducted each surgery. A patient was excluded if the individual met these criteria:

- Receiving ossiculoplasty or mastoidectomy, or
- Having unclear hearing threshold, such as “>110 dB”

For each patient, we carried out tympanoplasty under general anesthesia, denuded the perforation as routine, develop a tympanomeatal flap, and placed the grafting material under the tympanomeatal flap and the drum (underlay technique). In postauricular approach, we exposed the temporalis muscle to harvest a piece of temporal fascia. In transcanal approach, we made an up-down incision medial to the tragus, harvested a single piece of the anterior side perichondrium with some connective tissue, and preserved the cartilage. Both grafting materials were thinned and dried.

Because postoperative hearing might [26] or might not [27] be affected by age. We conducted a t test on age between the 2 groups. Another t test on preoperative air-bone gap was carried out to exclude differences of disease severity. We compared the mean gap change (preoperative vs. postoperative) between the postauricular and transcanal groups and took a t test to see if there was a significant difference. Individual air-bone gaps were plotted before and after the surgery to show the variety for both groups at each frequency: 0.5k, 1k, 2k, and 4k Hz. T tests were examined to see if the surgery improved the air-bone gap at each frequency for each group. We performed a one-way analysis of variance (ANOVA) on gap change as a function of frequency to see if postoperative gap changes were similar across frequencies. A pair-wise comparison using Fisher’s least significant difference test was calculated to show where a significance existed.

The experienced ear surgeon, Huang, learned the transcanal approach by doing. He did not participate a cadaver study or training course before the shift. To find a possible learning curve, we scatter plotted individual postoperative gap changes vs. the surgery dates and calculated the Spearman rank correlation. A chi-square test of failed vs. successful repair case numbers for each group was conducted to investigate whether the successful repair rate differs between the 2 groups. The statistical significance was all tested as α = 0.05.

### Ethical statements

The Institutional Review Board (IRB) of Chang Gung Medical Foundation, Taiwan approved the study methods and protocols (IRB number: 202001856B0). We performed the study in accordance with Good Clinical Practice and the applicable laws and regulations. As a retrospective cohort study, the IRB approved the waiver of the participants’ consent.

## Results

The postauricular group had 35 surgeries in 34 patients with 20 women and 14 men. There were 18 left ears and 17 right ears. The matched transcanal group had 35 surgeries in 35 patients with 21 women and 14 men. There were 24 left ears and 11 right ears. The age distribution of the postauricular group (with a mean of 53.7 years ± 1 standard deviation (SD) of 17.7 years) did not differ from the age distribution of the transcanal group (58.0 ±16.6 years), p = 0.3017. As mentioned in Instruction, we compared the underlying middle-ear pathologies representing in preoperative audiometric variables in the two groups. The preoperative mean bone-conducting thresholds in the postauricular group (29.0 ± 18.0 dB) did not differ from those thresholds in the transcanal group (30.4 ± 14.6 dB), p = 0.7316. The preoperative air-bone gaps in the postauricular group (21.2 ± 10.7 dB) did not differ from those gaps in the transcanal group (20.4 ± 11.2 dB), p = 0.7449.

**Fig 1:**
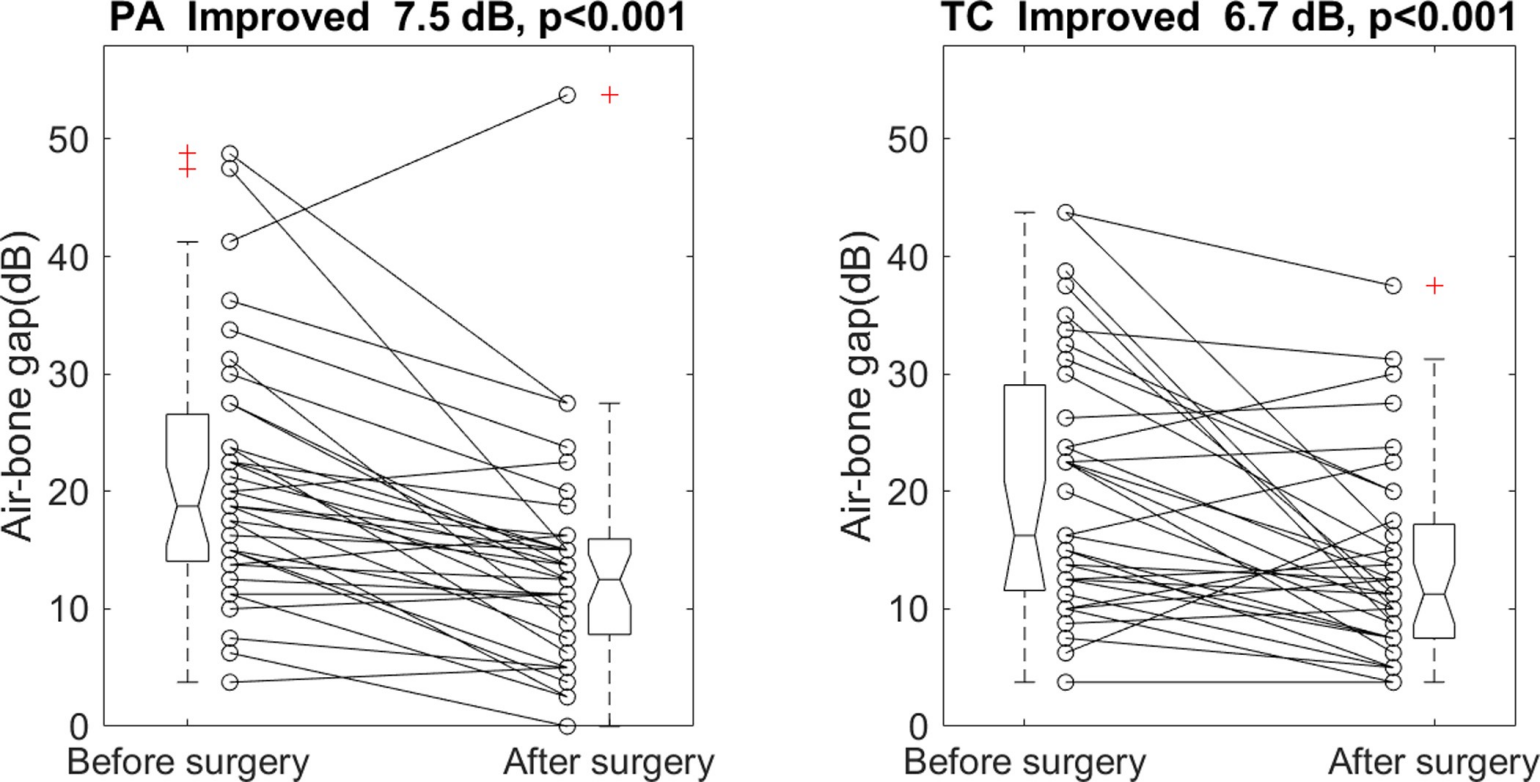
Individual mean air-bone gap of 0.5, 1, 2, and 4k Hz before and after the surgery. PA: Postauricular. TC: Transcanal. +: An outlier. Each boxplot displays a five-number summary: The minimum, the maximum, the median, and the first and third quartiles.

**Fig 2:**
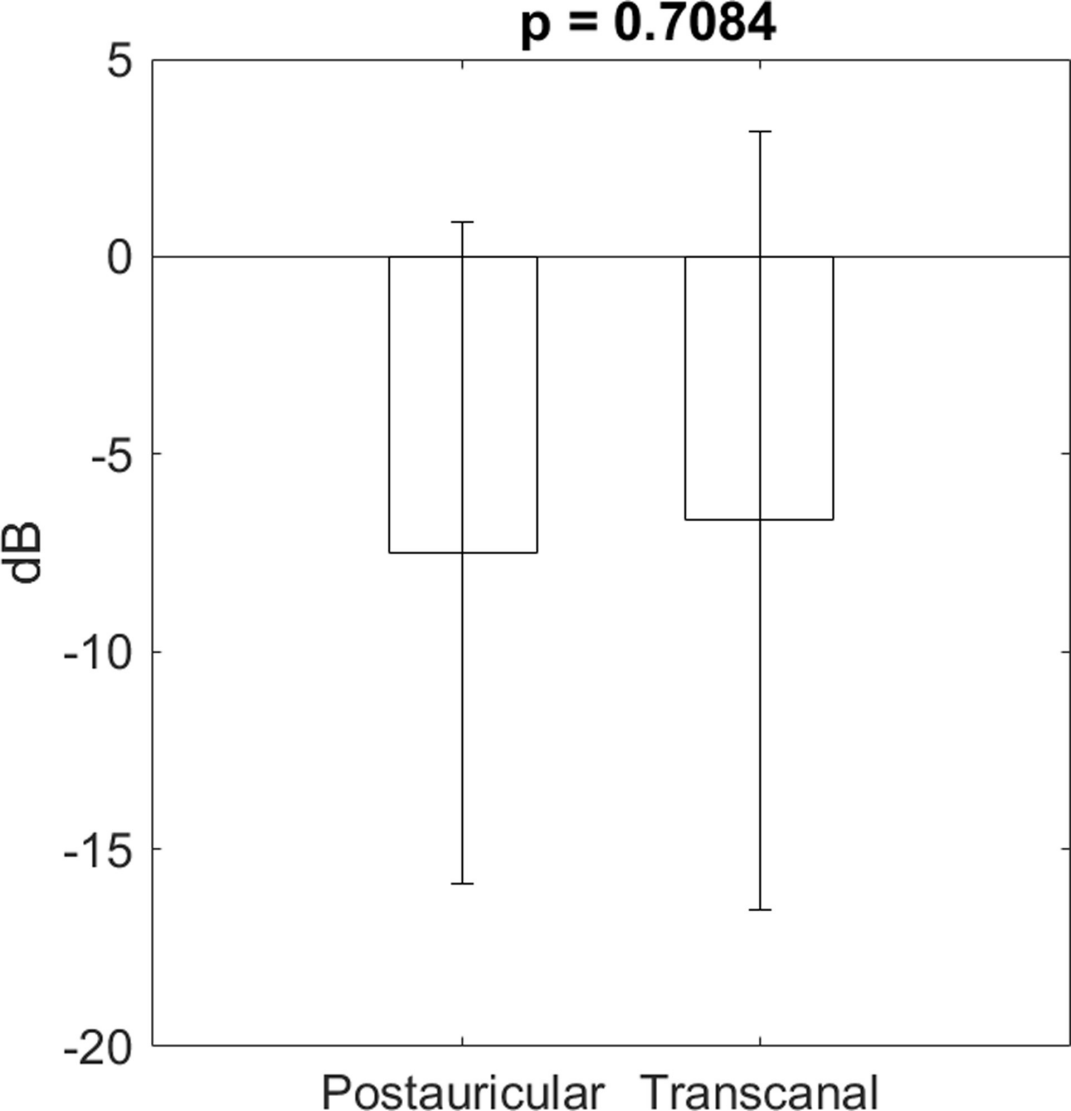
The mean changes of air-bone gap after the surgery in the postauricular group did not differ from those in the transcanal group.

A certified audiologist performed a hearing test 64 ± 43 days after the surgery in the postauricular group. The postauricular surgery reduced the mean air-bone gap of the 4 frequencies from 21.2 ± 10.7 dB to 13.7 ± 9.7 dB, p < 0.001. The mean air-bone gap improved 7.5 dB, with a 95% confidence interval of 4.6 to 10.4 dB. A hearing test was carried out 81 ± 57 days after the surgery in the transcanal group. The transcanal surgery reduced the mean air-bone gap from 20.4 ± 11.2 dB to 13.7 ± 8.4 dB, p < 0.001. The mean air-bone gap improved 6.7 dB, with a 95% confidence interval of 3.3 to 10.1 dB. Fig 1 shows the individual mean air-bone gap before and after the surgery in each group. The mean gap changes in the postauricular group (7.5 ± 8.4 dB) did not differ from those in the transcanal group (6.7 ± 9.8 dB), p = 0.7084 (Fig 2).

The postauricular surgery reduced the 0.5k Hz air-bone gap from 20.1 ± 15.5 dB to 8.9 ± 12.4 dB, p < 0.001. The mean 0.5k Hz air-bone gap improved 11.3 dB, with a 95% confidence interval of 6.9 to 15.7 dB. The transcanal surgery reduced the 0.5k Hz air-bone gap from 20.7 ± 14.9 dB to 11.6 ± 11.7 dB, p < 0.001. The mean 0.5k Hz air-bone gap improved 9.1 dB, with a 95% confidence interval of 4.0 to 14.3 dB.

The postauricular surgery reduced the 1k Hz air-bone gap from 25.7 ± 14.7 dB to 15.7 ± 12.3 dB, p < 0.001. The mean 1k Hz air-bone gap improved 10 dB, with a 95% confidence interval of 6.2 to 13.8 dB. The transcanal surgery reduced the 1k Hz air-bone gap from 24.0 ± 15.8 dB to 14.3 ± 11.3 dB, p < 0.001. The mean 1k Hz air-bone gap improved 9.7 dB, with a 95% confidence interval of 4.7 to 14.7 dB.

The postauricular surgery changed the 2k Hz air-bone gap from 17.9 ± 11.3 dB to 14.3 ± 10.7 dB, p = 0.082. It was not statistically significant. The mean 2k Hz air-bone gap improved 3.6 dB, with a 95% confidence interval of -0.5 to 7.6 dB. The transcanal surgery reduced the 2k Hz air-bone gap from 17.1 ± 8.9 dB to 10.4 ± 8.4 dB, p < 0.001. The mean 2k Hz air-bone gap improved 6.7 dB, with a 95% confidence interval of 3.8 to 9.6 dB.

The postauricular surgery reduced the 4k Hz air-bone gap from 21.1 ± 12.7 dB to 16.0 ± 10.6 dB, p = 0.029. The mean 4k Hz air-bone gap improved 5.1 dB, with a 95% confidence interval of 0.6 to 9.7 dB. The transcanal surgery changed the 4k Hz air-bone gap from 19.6 ± 12.3 dB to 18.4 ± 8.7 dB, p = 0.569. It was not statistically significant. The mean 4k Hz air-bone gap improved 1.1dB, with a 95% confidence interval of -2.9 to 5.2 dB.

**Fig 3:**
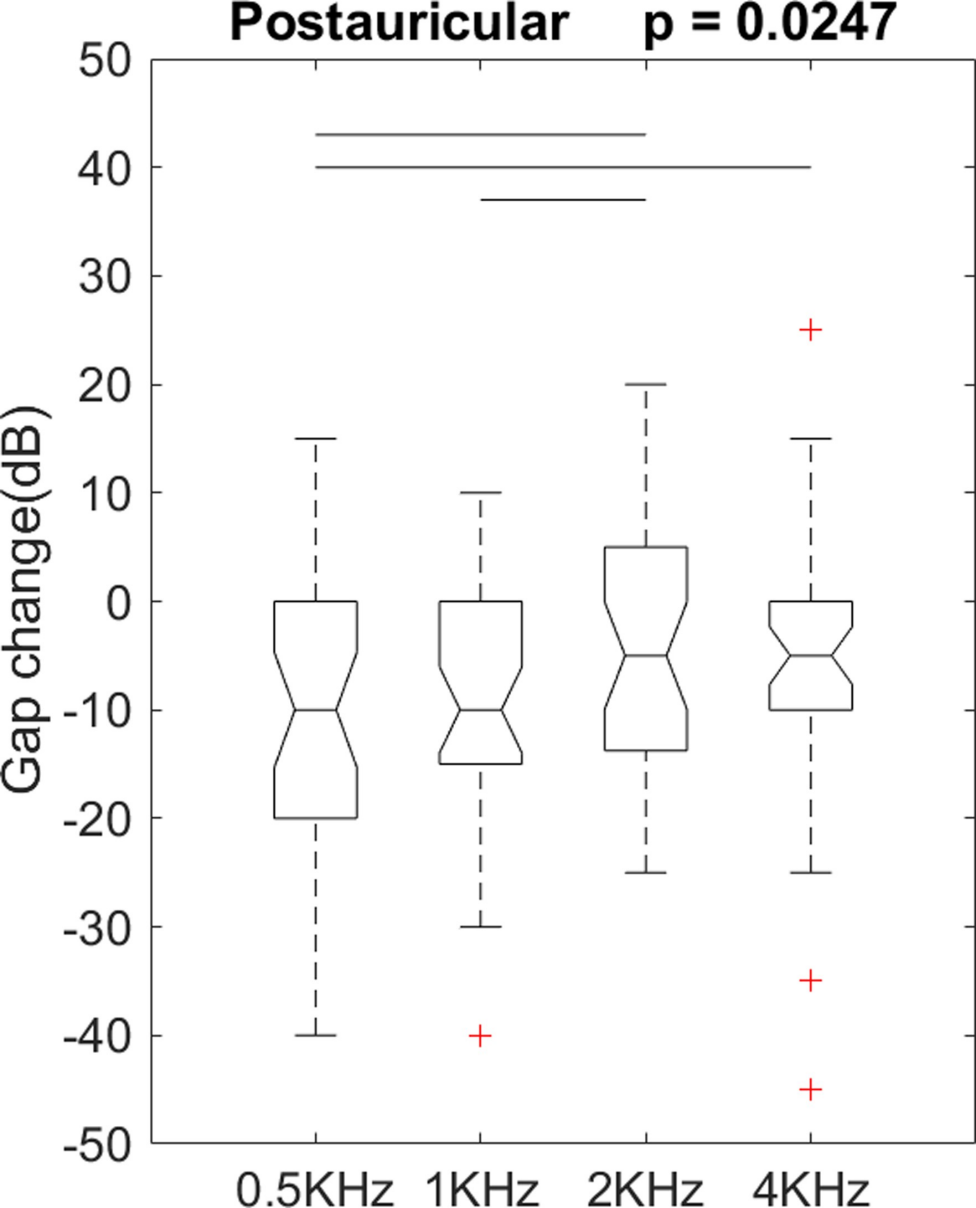
A one-way analysis of variance showed significantly different postoperative gap change among the frequencies in the postauricular group. +: An outlier. Each boxplot displays a five-number summary: The minimum, the maximum, the median, and the first and third quartiles. A horizontal line indicates a significant difference between the 2 frequencies in the post hoc comparisons.

For the postauricular group, we performed an analysis of variance (ANOVA) on gap change as a function of frequency to see if postoperative gap changes were similar across frequencies. There were 4 levels of frequency (0.5, 1, 2, and 4k Hz). There was a significant difference on postoperative gap changes among the levels of frequency, F(3, 136) = 3.22, p = 0.0247 (Fig 3). Post hoc comparisons using Fisher’s least significant difference test indicated differences between these frequencies: (0.5 and 2k Hz), (0.5 and 4k Hz), and (1k and 2k Hz), p = 0.0095, 0.0381, and 0.0301, respectively. There were no other significant differences on gap change among frequencies.

**Fig 4:**
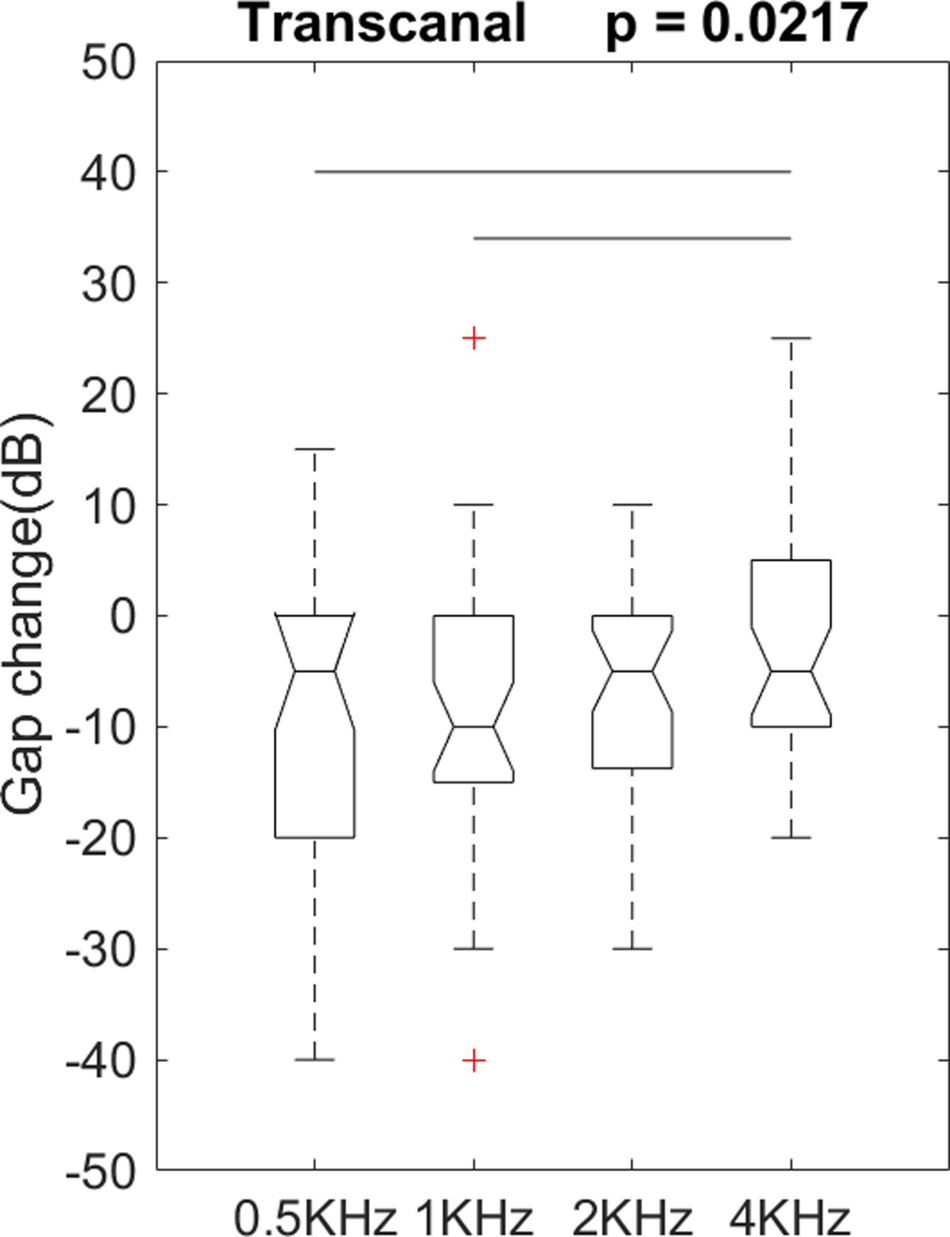
A one-way analysis of variance showed significantly different postoperative gap change among the frequencies in the transcanal group. +: An outlier. Each boxplot displays a five-number summary: The minimum, the maximum, the median, and the first and third quartiles. A horizontal line indicates a significant difference between the 2 frequencies in the post hoc comparisons.

For the transcanal group, we performed an ANOVA on gap change as a function of frequency (0.5, 1, 2, and 4k Hz). There was a significant difference on postoperative gap changes among the levels of frequency, F(3, 136) = 3.32, p = 0.0217 (Fig 4). Post hoc comparisons using Fisher’s least significant difference test indicated differences between these frequencies: (0.5k and 4k Hz) and (1k and 4k Hz), p = 0.0094 and 0.0055, respectively. There were no other significant differences on gap change among frequencies.

**Fig 5:**
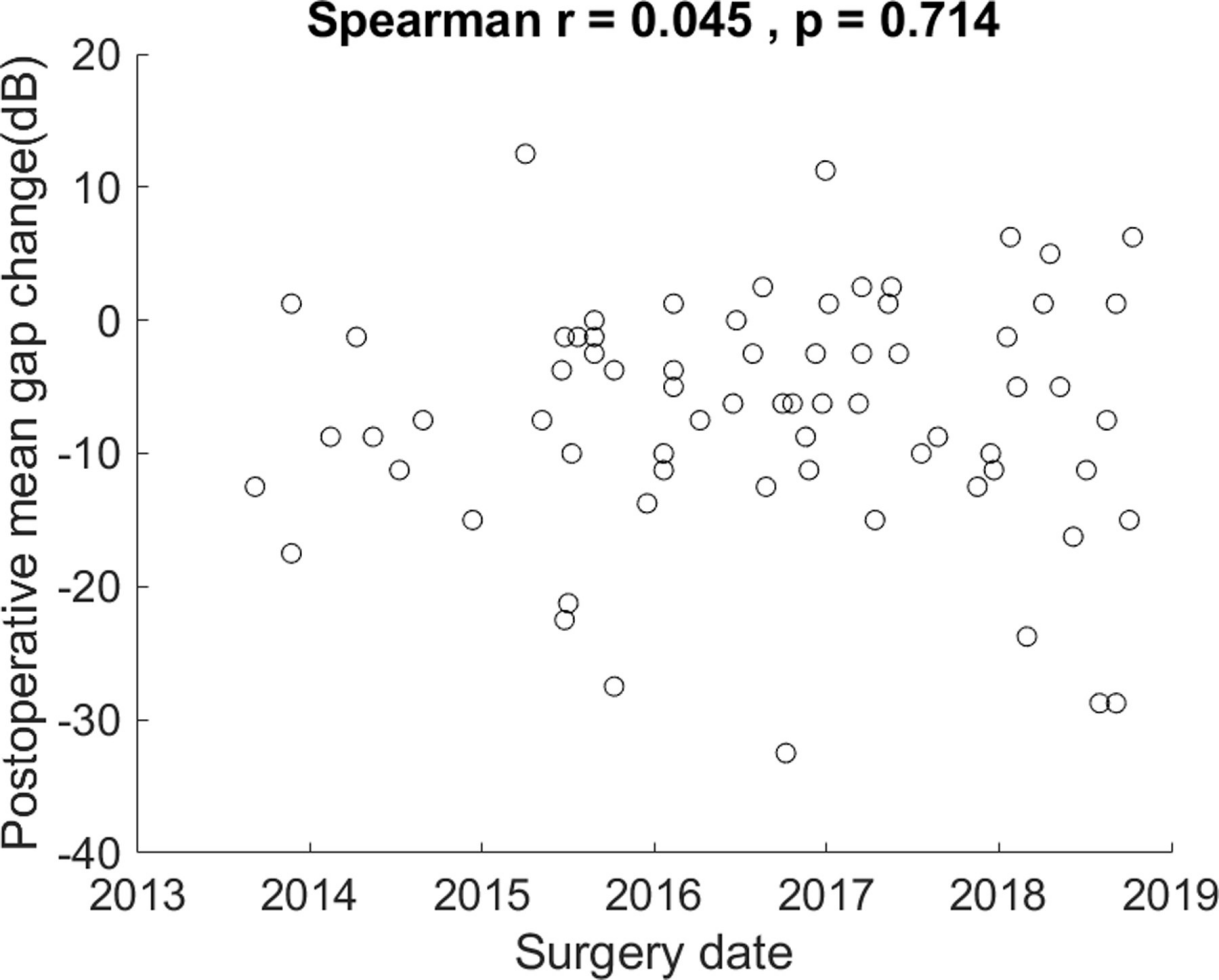
Scatter plot and Spearman rank correlation of postoperative mean gap change vs. surgery date. The non-significant relationship suggests a clinically non-significant learning curve.

A Spearman rank correlation for the postoperative mean gap change vs. surgery date revealed that mean gap change did not have a significant relationship with surgery date, r = 0.045, p = 0.714 (Fig 5).

There were 3 and 1 unsuccessful repair drums observed 3 months after the postauricular and transcanal surgeries, respectively. The successful repair rate was 91.4% and 97.1% after postauricular and transcanal tympanoplasties, respectively. A chi-square test showed the successful repair rate did not differ between the 2 groups, *X*^2^(1, N = 70) = 0.2652, p = 0.6066.

## Discussion

Unlike some previous studies comparing postauricular approach with a microscope and transcanal approach with an endoscope, we compared the outcomes using the same microscopic technique. With similar age and preoperative mean air-bone gap, the results show that both of postauricular and transcanal microscopic tympanoplasties reduced the mean air-bone gap and the gaps at 0.5k and 1k Hz after the surgery. Only the air-bone gap at 2k Hz in the postauricular group and that at 4k Hz in the transcanal group did not differ from the gaps after the surgeries. The ANOVA on gap change as a function of frequency (0.5, 1, 2, and 4k Hz) further show that both of postauricular and transcanal tympanoplasties resulted in a significant difference on postoperative gap changes among the levels of frequency. The post hoc comparisons display a common gap reduction difference between 0.5k and 4k Hz. These results together show that postauricular and transcanal microscopic tympanoplasties without ossiculoplasty have similar outcomes with better air-bone-gap closure at low frequencies. Kent et al. suggested that patients with high-frequency hearing loss due to drum perforation should not expect significant recovery from type-I tympanoplasty [28]. Our findings confirm Choi et al.’s report that the air-bone gap was primarily improved in the low and mid frequencies [14]. Ji and Zhai report similar results that the greatest improvement of air-bone-gap was found at 250 Hz [29].

On the change of grafting materials, our results show no different anatomical or functional outcomes between temporalis fascia or tragus perichondrium, in terms of the closure of mean air-bone gap and the difference of successful repair rate. This is far to conclude no effect of using temporalis fascia vs. tragus perichondrium due to the limitations and difficulties of study design, including control of blindness and learning curve. Similar results of no significant postoperative hearing difference were reported between fascia and perichondrium [6]. Although cartilage tympanoplasty might show a worse air-bone gap closure at high frequencies due to the graft’s stiffness or mass effect [4], several studies had reported comparable hearing outcomes between fascia and cartilage [30–33].

On the learning curve shifting from postauricular to transcanal approaches, there were few studies focusing on transcanal microscopic approach. Several studies investigated the learning curve shifting from postauricular microscopic to transcanal endoscopic tympanoplasty [1,9–11] or attic cholesteatoma surgery [34]. Studies diversely reported from a possible fast learning curve [9], comparable outcomes with gradually shortened operative times in 40 cases [34], a need of 50 endoscopic patients to achieve significant progress on successful repair rate [10], to approximately 60 operations to master the endoscopic technique [11]. Transcanal endoscopic approach may show a more favorable operative view in a learning process [35] with the proximity of the surgical filed and the approachability around the corner [34]. Our results from a single otolaryngologist that focus on microscopic approach show no correlation between the postoperative mean gap change and the surgery date (Fig 5), suggesting probable little learning-curve effect or a fast adoption of transcanal microscopic skills.

On the successful repair rate, Tseng et al. proposed that the comparable successful repair rate is related to the grafting technique (e.g., underlay grafting technique), rather than the different approaches (e.g., endoscopic transcanal or microscopic postauricular) [3]. We used underlay grafting in both postauricular and transcanal surgeries, and the results show the successful repair rate did not differ between the 2 groups.

## Conclusions

Rare studies reported frequency-specific hearing outcome with the learning curve for shifting from postauricular to transcanal microscopic tympanoplasty. The results show that both of postauricular and transcanal microscopic tympanoplasties reduced the mean air-bone gap, 0.5k Hz gap, and 1k Hz gap after the surgery. The further analyses on gap change as a function of frequency show that both of postauricular and transcanal tympanoplasties resulted in a significant difference on postoperative gap changes among the levels of frequency. The improvements show better air-bone-gap closure at low frequencies. We found the successful repair rate did not differ between the 2 groups. There was no correlation between the postoperative mean gap change and the surgery date, suggesting minimal learning-curve effect. The results of similar frequency-specific improvements and a minimal learning-curve effect may help to ease the concerns of those uncertainties before the shift.
